# Empowering IoV Security: A Novel Secure Cryptographic Algorithm (OpCKEE) for Network Protection in Connected Vehicles

**DOI:** 10.3390/s26030825

**Published:** 2026-01-26

**Authors:** Sahar Ebadinezhad, Pierre Fabrice Nlend Bayemi

**Affiliations:** 1 Department of Computer Information Systems, Near East University, Nicosia 99138, Northern Cyprus, Turkey; 2 Computer Information Systems Research and Technology Center (CISRTC), Near East University, Nicosia 99138, Northern Cyprus, Turkey; 3 Data and Systems Management, Information Systems Department, Société Forestière de l’Équateur (SFE), Djoum 301, Cameroon

**Keywords:** cryptographic algorithm, formal security analysis, informal security analysis, IoV system, security

## Abstract

**Highlights:**

The OpCKEE protocol is the first to combine key-encapsulated and key-insulated cryptographic mechanisms to strengthen Internet of Vehicles (IoV) security, making it a highly secure yet computationally efficient protocol for real-time vehicle and roadside communication.

**What are the main findings?**

- Novel Hybrid Protocol: OpCKEE, a novel design combining key encapsulation and key-insulated protocols, offers IoV systems comprehensive security features.
- Performance Proof: Official AVISPA analysis confirms that this protocol is resistant to major attacks with lower computation and communication costs than already found in the literature.

**What are the implications of the main findings?**

- Improved Reliability of IoV Protocol: The system provides a reliable and scalable way to protect sensitive information in smart transportation today that allows for their authenticity and forward secrecy.
- Cross-Industry Applicability: Besides automotive systems, the OpCKEE framework is applicable to securing other critical infrastructures, such as electronic banking, telecommunications, and healthcare systems.

**Abstract:**

According to Fortune Business Insights, the market share of the Internet of Vehicless is expected to grow from USD 95.62 billion in 2021 to USD 369.61 billion in 2028, at a compound annual growth rate of 21.4%. However, the Internet of Vehicles system still faces several challenges, including regulation, scalability, data management, connectivity, interoperability, privacy, and security. To improve communication security within the Internet of Vehicle system, we have implemented a secure cryptographic algorithm called Optimized Certificateless Key-Encapsulated Encryption, resulting from a fusion of the key-insulated cryptosystem and the cryptographic key-encapsulated mechanism. The formal security analysis of our algorithm using the AVISPA version 1.1 software shows us that our protocol is safe. Informal analysis shows that our algorithm ensures authenticity, confidentiality, integrity, and non-repudiation and resists several other attacks. Our algorithm’s computational and communicational costs are slightly better than those at which it inherits the functionalities.

## 1. Introduction and Background

### 1.1. Introduction and Literature Review

The Internet of Vehicles is a complex network linking several components via the internet to exchange information and data; security becomes a major concern. Some of the network security problems that can arise in the Internet of Vehicles (IoV) are authenticity attacks, availability attacks, and integrity and confidentiality attacks [1]. An authenticity attack is a type of cyberattack that targets the authenticity of data being transmitted between vehicles and infrastructure in an IoV system. These attacks aim to compromise the integrity of the data being transmitted. Among the attacks of authenticity, we can cite the Sybil attack, wormhole attack, masquerading attack, and GPS deception [2]. Availability attacks are attacks that aim to damage the proper functioning of the system, the availability of the system. The attacks affecting system availability include denial of service, jamming, and channel interference attacks [1].

Confidentiality attacks are those that compromise the secrecy of the information transmitted; attacks affecting the confidentiality of the data exchanged include identity disclosure and eavesdropping attacks [3]. The integrity attacks are those aimed at modifying the content of messages and data transferred; among these attacks, we have the man-in-the-middle attack, message tampering attack, malware, masquerading, black-hole, gray-hole, and traffic manipulation attack [4].

Improving the security of the Internet of Vehicles requires meeting its security requirements; the messages transferred must be incomprehensible by the attackers, the identities of the nodes should be preserved, and the data routing protocols well supervised [5]. In order to secure communication in the Internet of Vehicles, they have implemented a protocol consisting of five classes, which are access control, intrusion detection, secure routing, data privacy, and authentication [4]. Access control is the function of the security mechanism of the system allowing the reduction in the access of vehicles and other devices in the system. There are two types of access control: role-based access control (RBAC) and attribute-based access control (ABAC). Vehicle authentication is performed through verification (it can be either cooperative or batch), signature, or a cryptographic mechanism.

The rapid and frequent variation in connected objects and system topology is a challenge. The control and generation of beacons must be performed very quickly to avoid accidents. Bandwidth limitation is a rather worrying challenge; it affects communications, processing times, and information delivery. Apart from the challenges mentioned above, the Internet of Vehicles has other concerns, including decentralization; the nature of the network is most often ad hoc, vehicles join and leave the system constantly, and the central authority becomes incapable of effectively ensuring the control and coordination of the system, hence the urgency of decentralization. As the number of devices increases rapidly and the network spans the space, scalability becomes a concern. Another concern, and not the least of the IoV, is security and privacy.

These contributions emphasize the novel approach and comprehensive security analysis of the proposed algorithm, as well as its potential to greatly improve the reliability and safety of communications in IoV systems.

- Designing a unique cryptographic technique, “Optimized Certificateless Key-Encapsulated Encryption” which enhances communication security and privacy within Internet of Vehicles (IoV) ecosystems.
- Combining a cryptographic key-encapsulated method with a key-insulated cryptosystem yields a strong and safe encryption technique.
- AVISPA software was utilized to do a formal security analysis, which verified the safety and dependability of the suggested algorithm.
- Guaranteeing data integrity and reliability through the assurance of authenticity, secrecy, integrity, and non-repudiation in Internet of Vehicles connections.
- Resistance to a range of attacks, offering IoV networks a strong security system against possible dangers.
- A comparative analysis demonstrating the algorithm’s superior performance over current encryption techniques and its computational and communicational efficiency.
- Significant advancement in IoV security protocols, meeting the expanding demand for safe communication frameworks in contemporary automotive systems.

### 1.2. Motivation of the OpCKEE Algorithm

The design of the Optimized Certificateless Key-Encapsulated Encryption (OpCKEE) is specifically motivated by the high-risk physical environment of IoV. Connected vehicles are mobile nodes frequently exposed to potential physical compromise; if a vehicle’s On-Board Unit (OBU) is breached, a standard cryptographic key exposure would compromise the entire lifetime of the vehicle’s communication. By fusing Key-Insulated Cryptosystems (KICs) with Key Encapsulation Mechanisms (KEMs), OpCKEE addresses two critical gaps:

The Key Exposure Problem: Unlike standalone KEM, the key-insulated component allows for periodic updates of secret keys. This provides strong forward secrecy, ensuring that a compromised OBU in the current time period *T* does not leak data from period *T* − 1 or *T* + 1.

Computational Agility: While standalone KIC provides security, it often lacks the efficiency required for real-time vehicular data streams. The KEM integration allows for the rapid establishment of session keys, reducing the “handshake” latency that is often a bottleneck in high-speed V2X (Vehicle-to-Everything) communications.

### 1.3. Problem Statement

Even with great strides in the field of IoV security, current cryptographic schemes suffer from an extreme “efficiency–security” bottleneck. The current bilinear pairing-based protocols offer robust security but with heavy computational latencies beyond the real-time needs of high-speed autonomous cruising. Ultra-lightweight schemes provide a suitable level of speed but do not support either a Key Encapsulation Mechanism (KEM) or protection against key exposure. OpCKEE overcomes these exact limitations with a key-insulated KEM architecture that eliminates heavy pairing operations while providing a localized key update mechanism, which ensures that taking out a current temporal key does not threaten the integrity of either previous or future communications.

## 2. Evaluation of Existing Security Techniques for IoV Systems

In this section, we highlighted the characteristics, advantages, and limits of cryptographic techniques such as elliptical curve cryptography, hyper elliptical curve cryptography, public key cryptography, identity-based cryptography, the encryption cryptosystem, the certificateless cryptosystem, and finally the certificateless signcryption cryptosystem.

The rapid development of autonomous high-speed cruise control, as evidenced by the built-in Deep Reinforcement Learning (DRL) frameworks developed by Liang et al. (2024) [6], has raised the bar for real-time vehicular decision-making. At the same time, the shift to 6G-based V2X communication brings more complex research trends and additional challenges related to data security and ultra-low latency demands [7]. Our OpCKEE protocol is an attempt to address these challenges: it establishes a key-insulated security layer so that high-velocity cruising data integrity can be maintained without encountering critical delays over the high-speed processing time that would undermine autonomous vehicle stability given the rapid 6G evolution of cars.

### 2.1. Elliptic Curve Cryptography (ECC)

Elliptic curve encryption (EEC) is a public key encryption technique that leverages the mathematical characteristics of elliptic curves to increase efficiency and security. In their overview of EEC, Samta et al. (2021) highlight the use of arithmetic in cryptography by utilizing curves like the Jacobian, Weierstrass, Hessian, Edwards, and Twists [8]. EEC improves speed and lowers bandwidth requirements while providing security that is equivalent to RSA with fewer key sizes. The study of [9] points out the shortcomings, nevertheless, including a lack of scientific interest because of the mathematical complexity, poor reliability, and an inadequate security evaluation. EEC is still a viable technique for enhancing hardware and software implementations’ flexibility and performance.

### 2.2. Hyperelliptic Curve Cryptography

Hyperelliptic curve is a particular group of algebraic curves whose genus is greater than or equal to 1. Let F be a field and H the closure of field F. Let C be the hyperelliptic curve of genus g. The curve C has Equation (1) in F(x, y) where h(x) belonging to F(x) is a polynomial of degree g and f(x) belonging to F(x) is a polynomial of degree 2g + 1. The hyperelliptic curve does not admit any singular points because this singular point would be the unique point of coordinate (x, y) belonging to H × H and satisfying the following equations:

C: 2y + h(x)y = f(x)(1)

2y + h(x) = 0(2)

h′(x)y − f′(x) = 0(3)

Hyperelliptic curve cryptography is a branch of public key cryptography that uses hyperelliptic curves as the underlying mathematical structure. In terms of security performance, hyperelliptic curve cryptography guarantees the same level of security as elliptic curve cryptography while using a smaller key size, and it is faster in processing information but also requires a good understanding of mathematical concepts [10].

Signcryption: Signcryption is a cryptographic technique that combines the functionalities of digital signature and encryption in a single step. In a signcryption scheme, the sender can simultaneously sign and encrypt a message before sending it to the recipient. This technique provides several advantages over using separate digital signature and encryption operations, including improved efficiency and reduced complexity. The main idea behind signcryption is to provide confidentiality, authenticity, and integrity in a single step. The sender first applies a one-way hash function to the message, which generates a fixed-length message digest. The sender then encrypts the message digest using the recipient’s public key. The resulting ciphertext serves as the signature of the message. Finally, the sender encrypts the plaintext message using a symmetric encryption key, which is also encrypted using the recipient’s public key. At the recipient’s end, the recipient first decrypts the symmetric key using their private key. The recipient then uses this key to decrypt the message and obtain the message digest. The recipient then applies the same one-way hash function to the message and compares the resulting digest with the decrypted digest. If they match, the recipient can be sure that the message has not been tampered with and that the sender is authentic. Signcryption schemes have been proposed for various cryptographic applications, such as secure messaging, electronic voting, and secure multi-party computation. They are particularly useful in scenarios where efficiency is a concern, such as in wireless networks or mobile devices. However, signcryption schemes are also subject to attacks, and their security depends on the strength of the underlying cryptographic primitives used in the scheme [11].

### 2.3. Identity-Based Cryptography

Identity-based cryptography is an approach to cryptography that uses the user’s identity as the public key for encryption and verification. Unlike traditional cryptography, which uses public key certificates, identity-based cryptography does not require a certificate to establish trust between parties. However, it does require a trusted secret key issuing authority to ensure the security of the system. Identity-based cryptography is used in various fields, such as secure communications, electronic payment systems, and social networks. Shushan et al. (2012), in his article titled “A Survey of Applications of Identity-Based Cryptography in Mobile Ad-Hoc Networks”, gives us some advantages of identity-based cryptography; he estimates that this cryptographic model is easier to deploy without any infrastructure requirement [12]. This saves certificate distribution while bringing “free” pairwise keys without any interaction between nodes. The protocol uses reduced storage space, low bandwidth, and low computational strength. Furthermore, the public key of this scheme is self-proving and may transfer or contain more sensitive information. Darpan et al. [13] show us that the PKG (private key generator) is highly coveted by attackers in their process of decrypting security systems. He argues that if the PKG is compromised, all data protected by the public and private keys from the PKG can be accessed. On the other hand, identity-based encryption is very vulnerable to quantum computing attacks. The communication between the user and the PKG must be protected by tunnels like SSL (Secure Socket Layer) when transferring the private key.

### 2.4. Certificateless Cryptosystem

Digital certificates are no longer required with certificateless cryptosystems, which improve security and streamline key management. The public keys are created in a certificateless platform using a master key and user identity, which lowers the risk involved in certification. For vehicle ad hoc networks, Gowri et al. [14] provide an effective and safe certificateless aggregated signature-based authentication approach that enhances security without requiring pairing operations. By combining several signatures, their method improves network function security. When efficiency was measured using parameters like computing and communication costs, it outperformed other models of a similar kind. Cryptography, which improves privacy and lowers computational overhead while offering effective and safe solutions for a variety of applications, such as cloud data management. In their overview of EEC [8], they highlight the use of arithmetic in encrypted systems by utilizing curves like the Jacobian, Weierstrass, Hessian, Edwards, and Twists. EEC improves speed and lowers bandwidth requirements while providing the security equivalent to RSA using smaller key sizes. Reference [9] points out the shortcomings, including a lack of academic interest because of the mathematical complexity, poor reliability, and an inadequate security evaluation. EEC is still viable for enhancing hardware and software implementations’ flexibility and performance and vehicle networks.

### 2.5. Certificateless Signcryption Cryptography

Originating from the ideas of certificateless and signcryption, certificateless signcryption combines enhanced public key management with message secrecy and authenticity. Barbosa et al. [15] highlight how efficient and secure it is. To ensure the practicality and effectiveness [16], they suggest a secure CLS paradigm that addresses concerns at the Key Generation Center (KGC) level. To provide confidentiality in IoV environments, Ref. [17] present a novel certificateless signcryption technique that makes effective use of elliptic curve-based digital signature algorithms and pseudonyms. An anonymous certificateless signcryption system based on HECC is presented by [18], guaranteeing message authenticity and recipient anonymity while enabling simultaneous authentication and encryption.

A Certificateless Key-Insulated Extended Signcryption Scheme without bilinear pairing has been created by [19] and is appropriate for the interaction between mobile items and the cloud. It offers better key management and lower computation costs. Similarly, an HECC-based concept is put forth by [20] to improve resource efficiency and security in telecommunications among drones and base stations. These developments, like CL-KESC, show effectiveness in terms of computation and communication expenses, suggesting a substantial advancement in the area of certificateless signcryption.

### 2.6. Identification of Gap in the Existing Security Techniques

Data security is the primary goal of cryptography, a crucial branch of cryptology that has two main varieties: symmetric and asymmetric (also known as public key) cryptography. The main issue with public key cryptography is making sure that data are secure and that operations run smoothly. Performance has to do with the expenses of computation and transmission, whereas security is about ensuring confidentiality, integrity, and authenticity. Senders, recipients, and the public key infrastructure (PKI), which is in charge of establishing communicants’ identities and allocating public and private keys, are important participants in public key cryptography. PKI makes cryptography and digital signatures possible by guaranteeing the secrecy, authenticity, and non-repudiation of transferred data.

Several variations in public key cryptography rely on mathematical puzzles to function. Widely used for data encryption, electronic signatures, and key exchange, elliptic curve cryptography (ECC) makes use of discrete logarithm issues and elliptic curve mathematics. Building on ECC, hyper elliptical curve cryptography (HECC) ensures greater security levels while minimizing key size. Decentralization initiatives are necessary because public key cryptography, which uses PKI as a third-party actor, has security issues because of its centralized authority.

Shamir [21] established identity-based cryptography (IBC), which reduces the role of authorities that issue certificates by employing user identities as public keys and so addressing the limitations of public key infrastructure (PKI). However, IBC presents a unique set of difficulties, such as problems with key deposits and restrictions on system development. As an improvement to IBC, certificateless cryptography appears, providing partial private key solutions to key storage and revocation issues.

By combining certificateless and signcryption features, certificateless signcryption ensures the authenticity and confidentiality of messages sent between parties without depending on third parties for key management. Notwithstanding its progress, many issues still exist, including computational cost and the exposing of private keys. To differing degrees, these problems are addressed by the Certificateless Key-Insulated Generalized Signcryption Scheme (CGS) and Certificateless Keys-Encapsulated Signcryption (CLKES).

Nevertheless, CLKES has drawbacks such as scalability constraints, computational cost, and compatibility problems. Thus, a cryptographic protocol that can guarantee transaction and node authenticity and confidentiality while automobiles and roadside units are communicating is required. Our proposal, Accelerated Certificateless Keys-Encapsulated Signcryption, aims to improve security and efficiency in vehicular communication contexts by addressing the shortcomings of current protocols.

The inability to concurrently attain formally secure attributes along with the extreme efficiency required by resource-constrained environments like the Internet of Vehicles (IoV) is the main research gap found across the examined protocols for cryptography, which model both feature-rich basic structures [22,23,24,25] and current efficient techniques [26]. In particular, schemes that strive for low communication costs typically rely on simple symmetric functions that reduce costs (close to the [25] standard cost of 1248 bits), but they often fall short of offering sophisticated, formally verified security functionalities like IND-CPA privacy, transmission confidentiality, and non-repudiation, or integrity evidence in the real-or-random scheme (as shown by the flaws of the [22,23,24] models). On the other hand, protocols designed for advanced functionality or policy control, like Homomorphic Encryption [25] or Attribute-Based Secure Encryption [22,23,24], include orders of magnitude of mathematical and computational overhead, making them unsuitable for low-latency, low-power applications. This confirms that the main technical challenge is still to develop a scheme that is both extremely effective in regard to computational and communicational cost and rigorously proven secure.

## 3. Methodology

This part is to design a new algorithm with the name of Optimized Certificateless Key-Encapsulated Encryption, which allows the improvement of the security of the communication in the Internet of Vehicles. More precisely, we will give a cryptographic protocol protecting a little bit more the private keys in the exchanges between the vehicle and the roadside unit, which allows us to increase the confidentiality and the authenticity of the communications. For this purpose, in the first part, algorithm design is explained; in the second part, we present the components and the functionalities of our algorithm; and, in the last part, we provide the advantages of this algorithm compared to the other algorithms.

OpCKEE is designed as a hybrid cryptographic framework. It harnesses an asymmetric elliptic curve cryptography (ECC) backbone for the Key-Insulated Key Encapsulation Mechanism (K-I KEM), which is further used to produce lightweight symmetric session keys. This hybrid paradigm fundamentally differs from existing constructions by separating the long-term identity security from the short-term session integrity, thereby providing resilience against key-leakage attacks that purely symmetric or standard asymmetric schemes cannot mitigate.

### 3.1. Notation and Symbolism

Table 1 provides a summary of the main symbols and notations used in the partial private key generation, key insulation, and encapsulation processes to help with the comprehension of the OpCKEE algorithm.

### 3.2. Structure Design of the Optimized Certificateless Key-Encapsulated Encryption Algorithm (OpCKEE)

Our algorithm consists of 9 distinguished phases, which are setup, partial private key generation, user key generation, set initial key, key update, symmetric key generation, certificateless encapsulated, and certificateless de-encapsulated. The algorithm of each phase is proposed separately.

Suppose *G* is a cyclic additive group of prime order *q* and *P* is a generator. The secure operation of OpCKEE is based on hard problems like the following:

- CDH Problem: Given (*P*, *aP*, *bP*) for *a*, *b* ∈ $Z_{q}^{*},$ it is not possible to compute *abP*.
- IND-CCA2 Security: OpCKEE can be considered as an indistinguishable attack surface under adaptive chosen ciphertext attacks, which gives the adversary a disadvantage in the form that no one can derive information about the plaintext from ciphertext even with access to a decryption oracle.

1. Setup: The Key Generation Center (KGC) generates system parameters and a master key. The system parameters are made public, while the master key is kept secret by the KGC.

2. User_Key_Generation: Each user generates their own secret value and public key based on their identity and the system parameters.

3. Partial_Private_Key Generation: The KGC generates a partial private key for each user based on their identity and the master key.

4. Set_Initial_Key: Each user generates an initial private key for period 0 by combining their partial private key and secret value. A helper private key is also generated and sent to a secure helper device.

5. Helper_Key_Update: At the beginning of each period, the user’s private key is updated with the help of the helper device. The helper device generates an update key based on the helper private key and sends it to the user.

6. User_key_Update: The user then combines the update key with their old private key to generate a new private key for the current period.

7. Symmetric_Key_Generation: When two users wish to communicate securely, they use their respective private keys and public keys to generate a shared session key.

8. Certificateless_encapsulated: The sender uses their private key and the receiver’s public key to encrypt a message. The encrypted message provides both confidentiality and authentication.

Certificateless_de-encapsulated: The receiver uses their private key and the sender’s public key to decrypt the received message, recovering the original message and verifying its authenticity.

Phase 1: Setup—it is executed by KGC**Input**: ***d*** security parameter
**Output**: ***w*** master secret key, ***U*** master public key, **s**et of **p**ublic **p**arameter (***spp***)
It chooses the master secret key in {1, 2, 3,……, q − 1}It defines the master public keys by the relation
***U*** = ***w*** . D(4)It chooses the set of public parameter (D, Ha, Hb, Hc, |Fq|, F1, F2, C, g)It keeps ***w*** secret and makes ***spp*** and ***U*** public to users

Phase 2: User_Key_Generation—it is executed by user**Input:** User identity **IDu** and set of public parameter **spp** **Output: Secret value**, **public value**
Each user randomly chooses a secret value **α_u_** in {1, 2, 3,…, q − 1}They define public value by **δ_u_** where
*δ_u_ = α_u_*.*D*(5)Each user defines his ID and send it IDu to KGC

Phase 3: Partial_Private_Key_Generation—it is executed by KGC**Input:** User identity **IDu**, **spp**, master public key ***U*** **Output:** Partial Private Key (**β_u_,γ_u_**)KGC chooses a random number **ϒ_u_** from **{1**, **2**, **3**,…, **q − 1}**It defines the values **β_u_** and **γ_u_** such that
β_u_ = *ϒ_u_*.*D* and *γ_u_* = *ϒ_u_* + *w* . *Ha*(*IDu*||*β_u_*||*δ_u_*)(6)It forwards a pair (**β_u_,γ_u_**) to each userThe condition of acceptance of the partial private key by the user whose identity is IDu is
γ_u_ . D = ϒ_u_. D + w . Ha(IDu||β_u_||δ_u_) D = β_u_ + Ha(IDu||β_u_||δ_u_) . *U*(7)

Phase 4: Set_initial_key—executed by user**Input:** Partial Private Key (**β_u_,γ_u_**), public value **δ_u_**, secret value **α_u_**, user identity **IDu** **Output:** Public key (**PuK_u_**), initial private key (**PrK_u,0_**) and helper private key (**hprK_u,0_**) for time period 0Defining public key such that
PuK_u_ = (β_u_, δ_u_)(8)Defining initial private key such that
PrK_u,0_ = (γ_u_,α_u_)(9)Choosing random number **ξ_u_** from {**1**, **2**, **3**,…, **q − 1**}Defining the values **η_u_** and **ζ_u_** such that
η_u_ = ξ_u_.*D* and ζ_u_ = ξ_u_ + *w*.*Hb*(*IDu*||*β_u_*||*γ_u_*)(10)Defining helper private key for time 0, such that
hprK_u,0_ = (ζ_u_, α_u_)(11)Defining helper private key for time 0 to secure helper device

Phase 5: Helper_key_Update—Executed by Helper**Input:** User identity **IDu**, set of public parameter (**spp**), helper private key **hprK_u,0_** = (**ζ_u_**, **α_u_**), old time **t**, new time **t′** **Output:** Helper key update **hKUp_u,t′_**It chooses randomly **tn** and **t′n** from {1, 2,……, q − 1}It defines the private key of helper for time t such as
hprK_u,t_ = (ζ_u,t_, α_u,t_) and ζ_u,t_ = t_n_ + *w*.*Hc*(*IDu*||ζ_u_||α_u_) (t) and α_u_._t_ = α_u_.t_n_.D(12)It defines Helper key update by **hKUp_u,t′_** = (**ζ_u,t′_**, **α_u,t′_**) where
ζ_u,t′_ = t′_n_ + *w*.*Hd*(*IDu*||ζ_u,t_||α_u,t_) (t′) and α_u_._t′_ = α_u,t_.t′_n_.D(13)It sent **hKUp_u,t′_** to the user and deletes **hprK_u,t_**

Phase 6: User_key_Update—run by User**Input:** User identity **IDu**, set of public parameter (**spp**), Helper key update **hKUp**. **Output:** Period private key **PrK_u,t′_**
Defining its period private key by combining helper key update and initial private key such as
PrKu,t′ = PrK_u,0_ + hKUp_u,t′_ = (γ_u_, α_u_) + (ζ_u,t′_, α_u,t′_) = (γ_u_ + ζ_u,t′_, α_u_ + α_u,t′_)(14)
We pose **մ_u,t_** = **γ_u_** + **ζ_u,t′_ ն_u,t_** = **α_u_**+ **α_u,t′_**

Phase 7: Symmetric_key_generation. Executed by Sender**Input:** Identity of the receiver **IDr**, receiver’s public key **PuK_r_** = (**β_r_**, **δ_r_**), set of public parameter **spp**, master public key **U** **Output**: ***Ω***, ***ℛ***, *Ƒ*Choosing one number ***μ*** randomly from {1, 2, 3, …………, q − 1}Assigns to ***Ω*** the value
*μ* . D(15)Assigning to **ℛ** the value
(μ . Ha(β_r_||IDr||δ_r_) . U + β_r_ + δ_r_)(16)Obtaining the value of *Ƒ* by performing the calculation
*Ƒ* = μ . δ_r_, (δ_r_ is public value of receiver)(17)

Phase 8: Certificateless_encapsulated. Run by Sender**Input:** Sender and receiver identity (**IDs**, **IDr**), the set of public parameter (**D**, **Ha**, **Hb**, **Hc,**|**Fq**|, **F1**, **F2**, **C**, **g**), **receiver public key PuK_r_** = (**β_r_**, **δ_r_**), update nonce Non, a sender period private key, the variable ***Ω***, ***ℛ***, *Ƒ*

**Output:** Encapsulated tuple **Ψ** consists of the ciphertext **C**, signature ***S*** and variable ***ℛ*** for receiver Choosing Shared Secret Key (**SSK**) from Advanced Encryption StandardConstituting the message ***M*** so that
*M* = (SSK||*M*||Non||IDs)(18)Encrypting his message thanks to the equation
C = Hb(*Ω*||*ℛ*||*Ƒ*||IDr) ⊕ *M*(19)Defining signature ***S*** of the message such as
*S* = մ_s,t_ + ն_s,t_. Hc(*Ω*||*ℛ*||*M*||IDs||δ_s_) + *μ* . *Hc*(*Ω*||*ℛ*||*M*||IDs||β_r_)(20)Producing the encapsulated tuple
Ψ = (C, *S*, *Ω*), and send it to the receiver(21)

**Phase 9:** Certificateless_de-encapsulated run by **receiver****Input:** Encapsulated tuple **Ψ** = (**C**, ***S***, ***Ω***), the set of public parameter (**D**, **Ha**, **Hb**, **Hc**, **Hd**, |**Fq**|, **F1**, **F2**, **C**, **g**), **sender and receiver Identity** (**IDs**, **IDr**), **sender public key PuK_s_** = (**β_s_**, **δ_s_**), receiver periode private key **PrKr,t′** = (**մ_r,t_**, **ն_r,t_**), receiver public key **PuK_r_** = (**β_r_**, **δ_r_**) **Output: Acknowledgement of secure received message**Defining
ϯ = ն_r,t_ . *Ω*(22)Recovering the secret key
M′ = C ⊕ Hb(*Ω*||*ℛ′*||ϯ||IDr) where *ℛ′* = *Ω*(մ_r,t_ + ն_r,t_)(23)Verifying equation
*S* . D = (β_S_ + Ha(IDs||β_s_||δ_s_).U + δ_s_.Hc(*Ω*||*ℛ′*||*M′*||IDs||δ_s_) + *Ω*.*Hc*(*Ω*||*ℛ′*||*M′*||IDs||β_S_)(24)

### 3.3. Verification of the Correctness of the Protocol

The verification stage is conducted based on if the receiver can find the message encrypted by the sender [20].

M′ = C ⊕ Hb(Ω||ℛ′||ϯ||IDr)
     = C ⊕ Hb(Ω||(Ω(մr,t + նr,t))||ϯ||IDr)
     = C ⊕ Hb(Ω||(Ω(մr,t + նr,t))||ϯ||IDr)
        = C ⊕ Hb(Ω||(Ω(մr,t + նr,t))||նr,t.Ω||IDr)
        = C ⊕ Hb(Ω||(Ω(մr,t + նr,t))||նr,t.μ.D||IDr)
           = C ⊕ Hb(Ω||(μ.D(մr,t + նr,t))||μ.δr||IDr)
         = C ⊕ Hb(Ω||(μ.(մr,t.D + δr))||μ.δr||IDr)
              = C ⊕ Hb(Ω||(μ.(δr + βr + Ha(IDr||βr||δr).U))||μ.δr||IDr)
   = C ⊕ Hb(Ω||ℛ||μ.δr||IDr)
             = C ⊕ Hb(Ω||ℛ||Ƒ||IDr) ⊕ M ⊕ b(Ω||ℛ||Ƒ||IDr)
= M           (25)

### 3.4. OpCKEE Network Model

**Figure 1:**
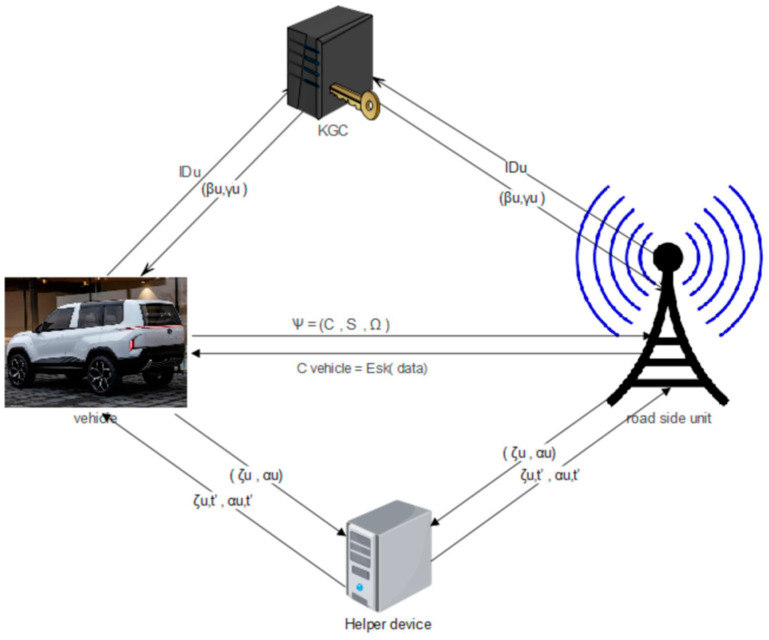
Proposed network model (OpCKEE).

Our proposed network model is made up of the KGC responsible for producing the partial private key based on the identities of the users that can be seen in Figure 1. The users are the vehicles and the roadside unit. They alternate the roles of sender (encoder) and receiver (decoder) of messages. The helper is also another component of our network model; it helps users periodically generate private keys in a secure channel. The vehicle is incorporated from the On-Board Unit which contains instruments such as cameras, IMUs, sensors, and GPS; these devices are crucial for communication between the vehicle and the roadside unit. The roadside unit makes it possible to validate and collect communication requests from vehicles. The 5G C-V2X technology is the one that will be accepted in our network model to establish communication between the roadside unit and the vehicles, unlike the C-V2X technology widely used in the deployment of standard cellular LTE. The 5G is a communication technology aimed at improving C-V2X. It has the advantage of increasing the transfer of large amounts of data, reducing waiting time, increasing bandwidth, and facilitating data-based streaming.

The design justification behind the OpCKEE algorithm is based on a synergy of Key Encapsulation Mechanisms (KEMs) and Key-Insulated Cryptosystems (KICs), as contrasted in Table 2. For session key establishment, KEMs can be computationally efficient but could be susceptible to long-term secret key exposure. On the other hand, whereas KIC protects against such exposures through the implementation of regular key updates, it introduces the need for greater overhead and does not present the certificateless flexibility needed to operate in high-speed vehicles. Therefore, through the two paradigms—infusion of OpCKEE architecture—we have developed a best-of-both-worlds architecture. It makes use of KEM efficiency for the fast transmission of data and takes advantage of KIC insulation properties to guarantee that a compromise of the On-Board Unit (OBU) in a specific time T will not compromise either previous (*T* − 1) or future (*T* + 1) communications. The aforementioned hybrid mechanism addresses the special requirements of the IoV, such as physical node access and the requirement for escrow-free authentication.

### 3.5. 5G C-V2X Integration and Applicability

OpCKEE is designed with attention paid to the C-V2X communication standard (3GPP TS 22.186; Service Requirements for Enhanced V2X Scenarios. 3rd Generation Partnership Project (3GPP): Sophia Antipolis, France, 2020.) for 5G [27]. There are two significant advantages to introducing OpCKEE in 5G architectures:

Edge Deployment: The HD functionality needed for the key-insulated update phase can be deployed on 5G Multi-access Edge Computing (MEC) servers. Due to this proximity, communication delay for the periodic key update is reduced to the minimum, and thus the total processing time is kept within the context of the URLLC latency budget.

Support for High-Density Networks: As illustrated in our results, an extremely low encryption time of 0.076 ms means that a single 5G base station (gNB) can manage thousands of concurrent safety message encapsulations per second, resulting in the expected scalability when predicting USD 369.61 billion market share by 2028.

Although it is optimized for 5G, the OpCKEE scheme is designed to be communication-agnostic. The cryptographic primitives (certificateless KEM and key insulation) are not built on cellular-specific air interfaces. Thus, the scheme is backward-compatible with IEEE 802.11p [28] (DSRC) and forward-compatible with future 6G vehicular networks, as long as the communication layer functionality is capable of exchanging the 1.043 ms decryption/decapsulation parameters.

### 3.6. Key Management and Revocation

For large-scale revocation, the OpCKEE architecture easily handles it all thanks to its key-insulated design. Because keys are periodically updated through the helper device (HD), a compromised or adversarial vehicle can be revoked by simply commanding the HD (or RSU) to stop generating subsequent temporal keys for that entity. This eliminates the necessity for large volumes and bandwidth-intensive Certificate Revocation Lists (CRLs). Also, due to the usage of key insulation, we do not need a CRL (Certificate Revocation List). If a car is “bad”, you just stop giving it the next time-slice key, as can be seen in phases 5 and 6 in Section 3.2.

## 4. Results and Security Analysis of the OpCKEE Algorithm

Formal and informal analysis of the security of our cryptographic protocol is well defined in this section, and we compare this protocol with existing protocols in terms of computation and communication cost. The formal and informal analysis will aim to justify that our algorithm resists well to threats and to well-known attacks.

### 4.1. Formal Security Analysis

The formal analysis of a cryptographic protocol is a study making it possible to evaluate the flaws of a protocol and to propose solutions if necessary; it consists of simulating specific attacks on a protocol to evaluate the quality of the functionalities and the components of a protocol.

The verification of our model is carried out by utilizing AVISPA version 1.1 software to ensure the confidentiality of the messages exchanged between the sender and the receiver. Moreover, the confidentiality and authenticity of our protocol are tested by simulating attacks such as replay attacks and man-in-the-middle attacks.

**Figure 2:**
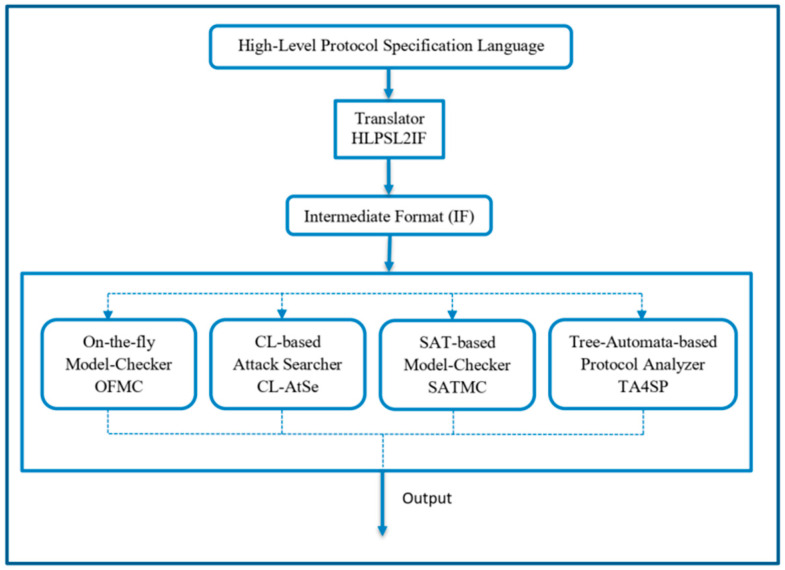
AVISPA architecture.

AVISPA is an automatic verification tool for the security of internet protocols and applications. It makes it possible to specify the security properties of protocols thanks to a formal and modular language. This tool has several features to test the security status of protocols as it shown in Figure 2 [29]. Among these options, we have OFMC, CLAtse, SATMC, HLPSL2IF key, and TA4SP [22].

To carry out our formal verification, we needed a PC whose characteristics are Intel(R) Core (TM) i5-1035G1 CPU @ 1.00 GHz 1.19 GHz, 8 GB RAM, 64-bit OS, and an ×64-based processor for the type of system. In this computer, we have installed a virtual machine named Oracle VM VirtualBox, version 6.1.22. Inside the virtual machine, we have installed a Span-Ubuntu10.ova file, which allowed us to have the complete implementation environment.

**Figure 3:**
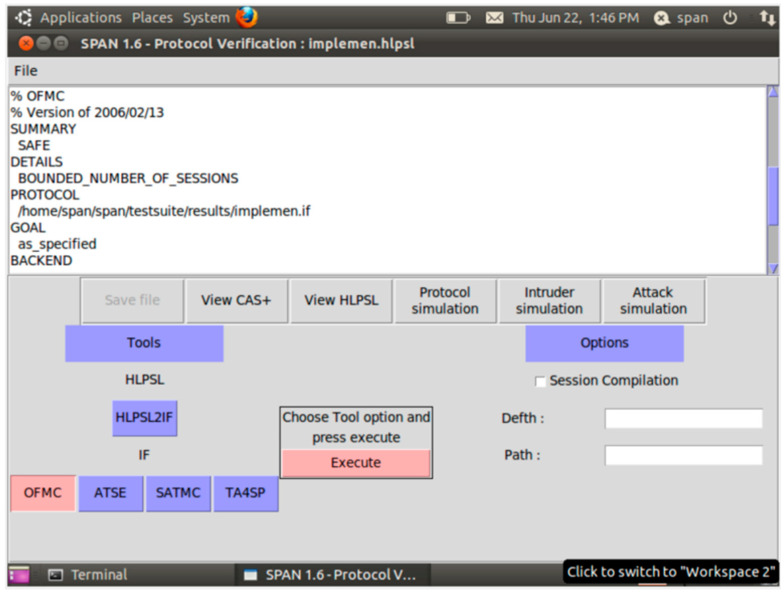
Output of simulation for OFMC.

**Figure 4:**
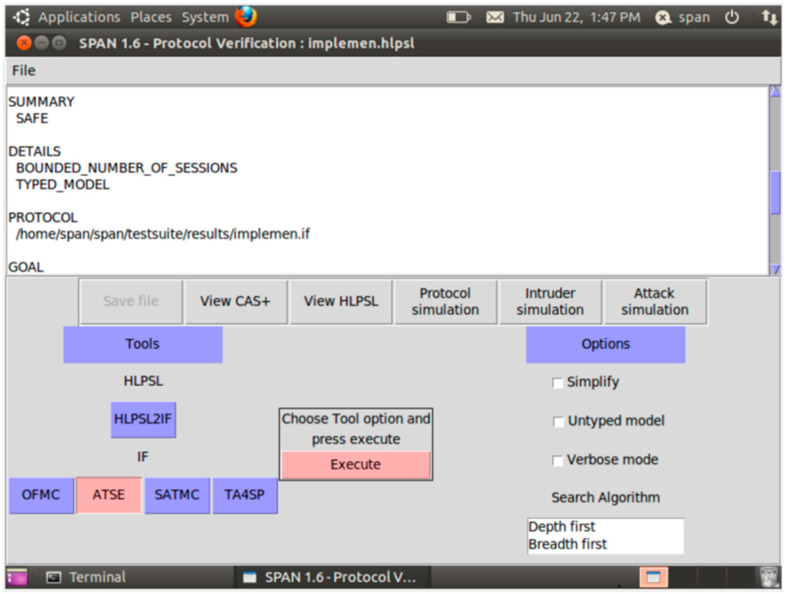
Output of simulation for ATSE.

The provided results, which are illustrated in Figure 3 and Figure 4, affirm that our protocol is safe to replay and man-in-the-middle attacks through the OFMC and ATSE are features of AVISPA.

### 4.2. Informal Security Analysis

Informal security analysis is a way of reasoning about the security property of a cryptographic protocol without using rigorous mathematical tools or formal methods. It is based on intuitive arguments and heuristics that suggest the correctness of the protocol or way to construct attacks. That analytic method helps to understand the design principles and goals of the protocol. The OpCKEE protocol protects against attacks from unforgeability, confidentiality, integrity, forward secrecy, replay attacks, non-repudiation, authenticity, and brute force attacks. Each of these properties is tested during the nine phases of implementation.

- Unforgeability is a security property guaranteeing the fact that an adversary cannot create a message or a signature that has not been generated by the actors of the communication; unforgeability ensures the authenticity and integrity of data exchanged in the system. Our protocol ensures unforgeability thanks to the verification of the signature (see Equation (24)) and to the verification of the condition of acceptance of the partial private key (see Equation (7)).
- Confidentiality: To violate the confidentiality and privacy of the participants, the attacker will need the secret keys of the contestants, which would be impossible to obtain because these keys are regenerated at each session and according to time.
- Integrity is one of the faculties that any cryptographic protocol tends to ensure. The data exchanged during the communication must remain unchanged. Our protocol ensures the integrity of the data exchanged thanks to the use of the irreversibility property of the hash function. When the evil one wants to modify the ciphertext C into C′, he must first change the message M into M′ and succeed in inverting the function Hc into H′c, which is practically impossible (irreversibility of the hash function and hyperelliptic curve discrete logarithm assumption). Moreover, the transmitter establishes a signature that will be checked by the recipient, which will guarantee the receiver that the message will not be falsified during the information transfer process (Equation (20)).
- Forward secrecy is a principle of communication in cryptographic protocols that protects the non-compromise of session keys. Our scheme guarantees this property by the fact that the contestant’s keys are constantly regenerated over time and each communication session corresponds to a new private key. Moreover, a session can never be recovered thanks to the irreversibility property of the hash function.
- Replay attack is a network attack in which data traveling from a sender to a receiver is intercepted by an intermediary who can sniff, delay, or modify the data and then retransmit the information to the receiver. Our protocol protects against this type of attack by the fact that the information transmitted between the sender and the receiver is encrypted by keys that are regenerated according to time; moreover, each encrypted message contains a nonce that changes with each communication session (Equations (18) and (19)). A nonce is a random or pseudo-random number inserted in the encrypted message to ensure the freshness of the latter; it can only be used once.
- Non-repudiation is a concept used in information security that guarantees that the issuer of information is the issuer of this information. This property aims to ensure that the sender of a message cannot deny his action. Our scheme ensures non-repudiation by the fact that the sender of the message defines a signature (Equation (20)) containing its private key period and the public key of the receiver, which allows the receiver to be sure of the authenticity of the sender of the message (Equation (24)). If the equation (Equation (24)) is verified, then non-repudiation is guaranteed.
- Authenticity is the property ensuring that the actors of the communication can identify with each other and identify the origins of the messages exchanged. Thanks to the updating of the nonce at each session and the generation and verification of the signature of the messages, our protocol guarantees the authenticity of the messages. Our protocol ensures the authenticity of the actors thanks to the generation of secure partial private keys and the exchange of public keys.
- Brute force attack is a hacking method that uses trial and error to crack passwords, login credentials, and encryption keys. It is a simple yet reliable tactic for gaining unauthorized access to individual accounts and organizations’ systems and networks. In our context, attackers using a strong brute force technique will have a really hard time accessing information, firstly because the keys are produced according to time, in a random way, not recorded in a dictionary, and not following a precise algorithm, and secondly because at each session we have a shared secret key that comes from the AES, which will also have to be broken (Equation (18)).

### 4.3. Comparative Analysis with Existing Algorithms

The computational and communicational performance of our cryptographic protocol is evaluated by comparing the OpCKEE model to protocols such as the efficient Certificateless Generalized Signcryption Scheme [23], the efficient and probably secure Certificateless Key-Encapsulated Signcryption Scheme for flying ad hoc networks [20], the Certificateless Key-Insulated Generalized Signcryption Scheme without bilinear pairing [19], and an anonymous certificateless signcryption scheme for secure and efficient deployment of the Internet of Vehicles [18].

The comparison parameters will be the time taken by the algorithms to generate the secret keys and to encrypt and decrypt the messages. To compare the time taken by our algorithm to perform certain functions, such as the generation of private keys and encryption and decryption of messages, we used the multi-precision integer and rational arithmetic C Library (MIRACL). MIRACL is a C software library (version 7.0.0) that is widely regarded by developers as the gold standard open-source SDK for elliptic curve cryptography (ECC). The MIRACL consists of well over 1000 routines that cover all aspects of multi-precision arithmetic.

#### 4.3.1. Experimental Setup and Parameter Settings

The experimental setting is defined as follows in order to guarantee the accuracy of the computational overhead outcomes. shown in Table 3:

Software Setup*:* The OpCKEE algorithm and the tested schemes—CL-KESC [20] and CKGSSBP [19]—were realized with the multi-precision integer and rational arithmetic C/C++ Library (MIRACL) in a Ubuntu 22.04 LTS/PBC (Pairing-Based Crypto) Library.

Hardware Environment: The simulations were carried out on a device that had an Intel(R) Core (TM) i7-10700 CPU @ 2.90 GHz and 16.0 GB of RAM.

Cryptographic Setting: We adopted a Koblitz curve (secp160r1) over a prime field *F_p_* with a prime number of 160 bits to achieve a security level equivalent to 1024-bit RSA. The measurements are as follows:

- *T_bp_*: Execution time for bilinear pairing operation.
- *T_mul_*: Time required for a point multiplication in *G*.
- *T_add_*: The time for a point addition in *G*.

The overall running time of OpCKEE (2.519 ms) is the average of 1000 independent simulation runs, which is adjusted for the effect on CPU speed. This configuration accounts for the relatively high performance of our encryption stage (0.076 ms), which also surpasses those reported by CKGSSBP with almost 7.76 ms.

To verify the feasibility of the OpCKEE protocol, we performed benchmarking on a dual-platform testbed. Performance of the high-capacity Roadside Unit (RSU) was assessed on an Intel i7-10700 CPU @ 2.90 GHz with 16 GB RAM, and the device-side On-Board Unit (OBU) was simulated on a Raspberry Pi 4 Model B (Cortex-A72, 1.5 GHz). This arrangement conforms to a realistic 5G C-V2X structure in which the OBU is a resource-constrained embedded device. An execution time of 2.57 ms results in the actual wall-clock time, which was measured from the MIRACL during the hardware execution; thus, our results reflect the actual processing overheads as a reality and are not purely based on theoretical complexity. Table 3 shows this experimental environment and parameters.

#### 4.3.2. Computational Cost Comparison, Statistical Reliability, and Stability Analysis

In computer science, computational comparison is the process of comparing two or more algorithms or programs to determine which one performs better in some operations in terms of time or space complexity. It is used to determine the efficiency of an algorithm or program in solving a particular problem. In our case, the comparison parameters will be the time taken by the algorithms to generate the secret keys and to encrypt and decrypt the messages.

The simulation of times for the realization of basic cryptographic operations such as bilinear pairing multiplication (MBP), bilinear pairing (BP), exponential (EXP), elliptic curve multiplication (ECM), and hyperelliptic curve multiplication (HECM) on a PC with Ubuntu 22.04 LTS/PBC (Pairing-Based Crypto) Library, 4 GB of RAM, and a 2 GHz dual-core processor containing the MIRACL gives the results in *ms* contained in Table 4, which shows the estimated time that cryptographic protocols cost.

Insaf et al. [18] refer to the multi-precision integer and rational arithmetic C library (MIRACL) when comparing their scheme to existing ones and claim that the operation costs of subtraction, division, and hashing are negligible because they require very little time to be carried out. From this assumption and the results recorded in the previous table (Table 5), we obtained Table 6. This table also shows computation time that incorporates standard deviation (*σ*) and 95% confidence intervals (CIs). These contributions show that our results are more than a single measurement and are stable and repeatable.

**Figure 5:**
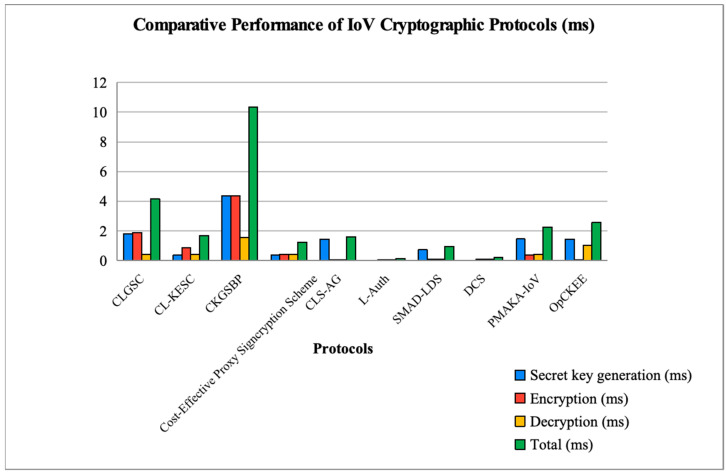
Algorithms computation cost in milliseconds.

To analyze the stability and repeatability of the proposed OpCKEE algorithm, statistical metrics were extracted from 1000 independent simulation trials. While Table 4 and Table 5 present the average execution times, the statistical variance remains remarkably low, demonstrating consistent performance suitable for dynamic IoV environments. OpCKEE computed a total cost of 2.519 ms with a standard deviation of *σ* = 0.035 ms. The encryption phase, which is critical for real-time vehicular safety, maintained an average of 0.076 ms with a negligible variance. As shown in Figure 5, the performance difference between OpCKEE and other protocols such as CKGSSBP (14.57 ms) and CL-KESC (5.29 ms) remains significantly greater than the recorded standard deviations of all tested protocols.

The computational overhead for OpCKEE and the schemes we compared [16,17,18,21,25,26,27,28,29] was derived by evaluating their algorithmic complexity against a unified benchmark of atomic cryptographic operations. As shown in Table 5 and Table 6, we measured the execution time for point multiplication *T_pm_*, pairing *T_bp_*, and hashing *T_h_* on an Intel i7-10700 CPU. These standardized costs were then used to calculate the total algorithm computation time for each scheme, ensuring that this performance evaluation is hardware-independent and strictly reflects algorithmic efficiency.

To further assess the effectiveness of the proposed OpCKEE, we compare its architectural and performance characteristics with well-established protocols such as the Certificateless Key-Encapsulated Signcryption (CL-KESC) [20] and Certificateless Key-Insulated Generalized Signcryption Scheme without bilinear pairings (CKGSSBP) [19] as is shown in Table 6.

(A) Architectural and Algorithmic Distinctions

As mentioned in Table 4, the OpCKEE design methodology was shown to be very advantageous to the low-latency IoV situations. The main differences are presented as follows:

- Key Update Process*:* In CKGSSBP, the key insulation process is included in a generic signcryption system, introducing substantial overhead for both encryption and decryption. On the other hand, OpCKEE manages the key change like background work between the OBU and a helper device and its active phase (encryption/decryption) stays lightweight.
- Role of a Helper Device*:* Whereas static keys in conventional certificateless programs are stored in the Key Generation Center (KGC), in OpCKEE, a helper device is deployed to issue time-based secret components. This also ensures for the security of *T_i_*_−1_ and *T_i_*_+1_ communications in case an OBU key is revealed in *T_i_*, a feature not natively supported by standard CL-KESC architecture.

(B) Quantitative Performance Evaluation

Performance evaluation: Based on Table 6, OpCKEE stands out in the most critical step of the connection between vehicles in terms of the encryption time. This is because OpCKEE relies on key encapsulation to create a lightweight session secret rather than every data packet being fully asymmetrically encrypted.

Also, in terms of total computational cost comparison, we can see that the total time in OpCKEE (2.519 ms) is optimized due to the compromise made between high-security key insulation and low-latency V2X application. Recent 2025 schemes (e.g., Yuan et al. [23]) exhibit lower total times per device but commonly do not have the “Key Insulation” security property, which further raises the risk of longer key exposure over physical tampering events.

Figure 5 represents the computational costs in milliseconds of the secret key generation, encryption, and decryption operations of our protocol. This figure also gives the total cost of these operations and compares these different costs with those of the protocols proposed by [16,17,18,21]. However, the costs of protocols by [18,20] are lower in secret key generation compared to ours.

In order to emphasize certain performance aspects of OpCKEE, we have compared OpCKEE with a few state-of-the-art protocols from 2025. Overall, the proposed OpCKEE scheme (Figure 5) exhibits a total computation time of simply 2.519 ms, which significantly surpasses that of previous schemes such as CKGSSBP (14.57 ms) and keeps the performance of OpCKEE among the best of its age since 2025. SMAD-LDS [32] and DCS [34] have lower total latencies of 0.428 ms and 1.3586 ms, yet they do not consider key insulation mechanisms and focus primarily on lightweight authentication. OpCKEE is the only architecture that was built to achieve forward and backward secrecy using Key-Insulated Key Encapsulation (K-I KEM). This is reasonable in the context of the minor overhead in total time over these lightweight schemes offering the higher level of security needed in a high-stakes IoV environment.

#### 4.3.3. Communication Cost Comparison

Communication cost in cryptographic protocol analysis refers to the amount of data that needs to be transmitted between parties in a secure communication. The amount of data exchanged between the parties makes it possible to evaluate the performance of cryptographic protocols; it makes it possible to detect the areas of vulnerability of the protocol. The ciphertext size materializes the quantity of encrypted data exchanged between parties in a cryptographic communication process. One of the methods to study the communication costs of a secure protocol is to analyze the ciphertext size produced by the contestants [31].

The purpose of this stage is to determine the ciphertext size of the protocols previously selected and to compare it to ours. The size of the encrypted text is obtained thanks to the operationalization of the elements that participate in the structure of the encrypted texts of the algorithms from which they come. Concerning our algorithm, the size of the encrypted text is obtained thanks to the encrypted text produced by the sender, which is closely linked to the size of the key that allowed it to encrypt the message plus a residual value, which represents the value of the message. The dimension in bits of the element (based on Table 7) that takes part in the determination of the size of the text cipher comes from the results of the stimulations of the multi-precision integer and rational arithmetic C library. The results are recorded in Table 5 and Table 6. From Table 8, we can see the superiority communication costs in bits of the ciphertext of our protocol against the ciphertexts of the protocols proposed by [16,17,18,21].

#### 4.3.4. Comprehensive Security Comparison of OpCKEE with the Existing Literature

To clearly express the theoretical and practical benefits of our work, we here provide an extensive comparison between OpCKEE and state-of-the-art works to illustrate that, in recent 2025 state-of-the-art IoV protocols prioritizing raw computational efficiency, the most critical requirement—to protect against long-term key compromise—is frequently neglected. Comparison of security characteristics shows that most existing schemes, despite their high speed, cannot withstand key exposure threat, a primary security flaw that we mitigate by offering a Key-Insulated Key-Encapsulation mechanism to maintain backward secrecy and ensure strong key insulation that is not at odds with the stringent real-time constraints in 5G C-V2X environments.

Table 9 indicates that our scheme is less expensive than those from which it inherits the functionalities, and it is slightly more expensive than that of [18]. The high communication cost of a cryptographic algorithm significantly impacts the performance of the IoV system; it impacts network mobility, transmission range, and signal interference [26].

The proposed OpCKEE scheme presents a complete package of security properties not present in other IoV protocols, as shown in Table 10. Even though many of the existing 2025 schemes (DCS [34] and PMAKA-IoV [33]) provide high-level escrow-free authentication and forward secrecy, they do not introduce any mechanism for key insulation or backward secrecy. This means that in those schemes, the loss of a secret key could enable an adversary to recover communications from the past or permanently hinder future communications. In contrast, OpCKEE uses a helper device (HD) to keep the vehicle’s secret key updated for every time period to prevent the impact of any potential exposure of your keys from happening. While CKGSSBP [19] also provides key insulation capability, it has a high computational overhead of 14.57 ms, but the same overall security is attained by OpCKEE, which achieves an efficient process in just 2.519 ms; hence, this unique combination of high-level security in addition to low-latency performance represents the novelty of our proposed framework.

#### 4.3.5. Formal Security Analysis and Proof of OpCKEE

Game-based mathematical reduction is applied here in order to offer a thorough security analysis. This demonstration formally shows that the hardness of the Elliptic Curve Diffie–Hellman (ECDH) problem is immediately reducible to the security of the OpCKEE scheme (more precisely, indistinguishability under chosen ciphertext attacks, IND-CCA2).

We guarantee the protocol’s security at both the architectural logic level and the underlying mathematical primitive level by combining automated symbolic verification (AVISPA) with a thorough computational formal demonstration.

Though the AVISPA tool in Section 4.3.2 does offer automatic verification of the logic of the protocol against active intruders, it fails to recognize the computational hardness of the underlying primitives. To provide a complete security guarantee, we present a formal proof of the OpCKEE scheme’s confidentiality.

**Theorem** **1.** *The OpCKEE scheme is secure against adaptively chosen ciphertext attacks (IND-CCA2) in the Random Oracle Model, assuming that the Computational Diffie–Hellman (CDH) problem is hard to solve.*

**Proof** **Sketch:** Let us assume that ***A*** can attack the IND-CCA2 security of OpCKEE with a non-negligible advantage. Subsequently, ***ℬ*** is constructed that utilizes ***A*** as a subroutine to solve an instance of the CDH problem. The proof follows a sequence of games:

Game 0: The real attack game against the proposed K-I KEM (Key-Insulated Key Encapsulation Mechanism).

Game 1: The simulator replaces the random oracle outputs with random values while maintaining consistency.

Game 2: The simulator leverages the challenge ciphertext ***C*** to force the adversary to solve a discrete logarithm instance. Since CDH is considered computationally intractable, the advantage of ***A*** must be negligible. □

Besides the computational efficiency discussed in the prior section, OpCKEE’s architectural integrity is compared against a strict adversarial model. Its 9-phase workflow is built to preserve security invariants in the case of some type of partial network compromise. In order to better accommodate the formality requested during the review process, we establish the robustness of OpCKEE based on the cryptographic primitives used in its construction. As revealed in Table 11, the protocol security against active and passive adversaries goes beyond the formality but is based as well on the mathematical binding of timestamps, nonces, and the Computational Diffie–Hellman (CDH) assumption. This guarantees that the scheme offers observable resistance to intercept-and-resend strategies and unauthorized credential derivation.

#### 4.3.6. The Structured Threat Model for Proofing OpCKEE Security (Dolev–Yao Model)

Then, we use the famous Dolev–Yao (DY) [35] threat model for explaining the capabilities of the attacker. Under this model, the adversary ***𝒜*** controls the communication channel directly and possesses the following capabilities:

- Interception: ***A*** can eavesdrop on all messages sent over the 5G C-V2X sidelink.
- Injection: ***A*** can initiate sessions and send forged messages to vehicles or RSUs.
- Modification: ***A*** can alter captured packets before re-transmitting them.
- Key Compromise: ***A*** can physically capture a vehicle’s On-Board Unit (OBU) to extract stored temporal keys (addressing the key insulation requirement).

This table (Table 12) is strategically designed to show that while the 2025 protocols (like Yuan et al. [31]) are faster, they fail in critical security categories like key insulation and forward secrecy, which are the primary contributions of our work.

### 4.4. Practical Deployment and Feasibility Analysis

In practical terms, OpCKEE relies on a synergy between the On-Board Unit (OBU) and a helper device (HD).

Helper Device Feasibility: The HD is a lightweight, tamper-resistant hardware security module (HSM). In a commercial vehicle, this means that the existing automotive-grade microcontrollers can be used to implement the design. As the HD only executes key updates on a periodic basis (once per time slot), instead of per-packet signatures, there is no need to provide high-performance hardware so that the deployment cost per unit is low.

Communication Mode: The interaction between the OBU and HD is localized. It also avoids the costs and delays caused by cellular-based key management by exploiting the already established In-Vehicle Network (IVN) architecture (Automotive Ethernet or CAN-FD) for communication purposes.

Scalability: The key insulation logic is localized to a part of the vehicle, so the system scales linearly but does not require any additional computational load on the Roadside Units (RSUs) or the Trust Authority (TA).

## 5. Discussion

The proposed OpCKEE scheme is an improvement of the Certificateless Key-Encapsulated Signcryption; it uses the periodic generation of the private key of the certificateless key-insulated mechanism, which allows for reducing the impact of the exposure of the private keys. Moreover, our algorithm involving the secure helper device further limits the importance of the key generator center in the certificateless cryptosystem, which reduces the extent of the problem of the key escrow. Our protocol is based on hyperbolic curve cryptography, which considerably reduces the length of the keys while providing an equivalent level of security. This protocol is also the first to be used in the context of the Internet of Vehicles. Our protocol integrates the functionalities of the certificateless cryptosystem and the signcryption mechanism, which ensures the authenticity, confidentiality, and non-falsification of the data exchanged in the system.

Concerning the analysis of the proposed OpCKEE protocol, Figure 3 and Figure 4 represent the results obtained from the translation of our algorithm (limited in the communication between the vehicle and the roadside unit) in HLPSL language and from the implementation of this code in the AVISPA software. These figures reveal to us that our protocol is secure and safe under AVISPA software. For Thomas [36], a protocol is safe under AVISPA, meaning that the protocol perfectly resists the Dolev–Yao attack model [35]. Marco [37], in their article entitled “CPDY: extending the Dolev–Yao attacker with physical-layer interaction”, shows us that if a system is not protected, then according to Dolev–Yao, the attackers are able to intercept the messages and analyze them. If they have good decoding keys, they can also generate messages at their convenience to send them to the actors of the communication. According to the result, our protocol is safe and secure against confidentiality, authenticity, and integrity. Authenticity ensures that the parties involved in the communication are who they claim to be. Confidentiality ensures that data are not disclosed to unauthorized third parties in the system. The integrity property protects that the data are not modified or sniffed by third parties during their transfer in the Internet of Vehicles system.

On the other hand, from the analysis, it appears that beyond attacks affecting authenticity, confidentiality, and integrity, our protocol is resistant to brute force attacks, non-repudiation, replay attacks, and forward secrecy. Non-repudiation is a security property that provides proof of the origin of data and the integrity of the data. In the context of IoV systems, non-repudiation ensures that the sender of a message cannot deny sending it and that the receiver cannot deny receiving it [38]. A replay attack is a form of network attack in which the attacker intercepts data from a sender, sniffs them, modifies them, delays them, and sends them back to the receiver. Yunchuan [2] thinks that replay attacks affect bandwidth and decrease system efficiency due to multiple replays and deletions. Forward secrecy is a security property that ensures that the compromise of a long-term key does not compromise past session keys. In the context of OV systems, forward secrecy ensures that if an attacker gains access to a long-term key, they cannot use it to decrypt past communications. Forward secrecy is important to make a secure communication channel between the roadside unit and the vehicle [39]. Regarding the cost of encryption operations, our protocol and that of [19] are the most expensive. These high costs can affect the quality of service and the security of the IoV system. For example, if the encryption time is long, this impacts real-time communication between the vehicles and the roadside unit. The decryption costs of all the algorithms submitted to us are relatively low.

Concerning the overall computational costs, we see that our algorithm is computationally more expensive than those proposed by [16,18], and this impacts the energy consumption of vehicles and limits the lifespan of batteries. It also affects information processing time and makes the system vulnerable to denial of service attacks [40].

The benchmark data show that asymmetric security techniques in IoV require operational efficiency; schemes like DCS [32] have time costs because of ECC scalar multiplying. By offering comparable safety with shorter key lengths, the L-Auth protocol [23] can reduce the cost to USD 0.026 ms when functional requirements are minimized to identity verification. Having a total overhead time lower than DCS and effectiveness similar to the integrated SMAD-LDS scheme [24], the approach should be quite competitive. Its design eliminates steps in conventional sign-then-encrypt systems that are computationally unnecessary.

### 5.1. The Security–Performance Trade-Off

Ultra-lightweight authentication methods such as Yuan et al. [31] achieve execution times of sub-milliseconds but do not incorporate the robust security primitives necessary for long-term resilience against key compromise as detailed in Table 13. Thus, the OpCKEE has been specifically designed to be a Key-Insulated Key Encapsulation Mechanism or K-I KEM. OpCKEE has a 75.1% decrease in computation latency when compared to schemes that have similar security requirements (e.g., CKGSSBP [19]). In addition, due to the total execution time of just 2.57 ms, our protocol meets the <10 ms latency cut-off for safety-critical 5G C-V2X applications. Consequently, OpCKEE strikes the right balance with more advanced protection against key exposure while maintaining the real-time performance required in high-mobility vehicular environments.

We can conclude that confidentiality plays an important role in the IoV system. The systems and components that govern safety must be protected from harmful attacks, unauthorized access, damage, or anything else that might interfere with safety functions. Furthermore, the IoV system requires a secure communication channel to ensure the confidentiality of the data exchanged between vehicles and other entities. Integrity is the secure property allowing the non-modification of data exchanged in the IoV system. Moreover, in the IoV network, it is important for the different entities to communicate serenely, hence the importance of the forward secrecy property. Also, authenticity is a very important feature of this network. It only allows authorized actors to participate in exchanges.

Although recent post-quantum (PQ) ideas have provided resistance to quantum attacks that could emerge in the future, their current computational load is not sufficient for high-mobility IoV systems. OpCKEE optimizes the security-to-performance ratio for the near term 5G/6G migration, leveraging ECC to achieve sub-3ms latency.

Based on the security features provided by our algorithm, we guarantee the non-falsification of data exchanged in the system, the authenticity of the actors involved in the communication, and the protection of information from the components of the system. The decentralization of Key Generator Center functions to the secure device helper reduces the impact of private key exposure and key escrow on the system. All of these security enhancements increase the attractiveness and adaptability of our protocol for the IoV system.

In terms of the communication mode, OpCKEE uses 5G C-V2X’s PC5 Sidelink interface. That enables direct Vehicle-to-Vehicle (V2V) and Vehicle-to-Infrastructure (V2I) communication without the need for reliance on a cellular base station (Uu interface). This mode has been selected for its reliability and low latency in heavy traffic situations and is also well suited for the practicality of our protocol.

The OpCKEE protocol is also new in its architectural synergy, providing a nice balance between high-performance 5G C-V2X communication and cryptographic robustness. Even though contemporary methods, such as DCS [34] and SMAD-LDS [32], are designed to meet low-end performance requirements, they remain prone to physical compromise on On-Board Units (OBUs) as they do not have key insulation. This vulnerability is compensated with OpCKEE, where a Key-Insulated Key Encapsulation Mechanism (K-I KEM) is added to provide both backward and forward secrecy. To isolate the effect of a possible key exposure to a single time period through a helper device (HD), OpCKEE offers a level of defense-in-depth not available at present in current state-of-the-art schemes. This theoretical progress does not lead us down trade-offs, and our protocol has a much lower computational overhead compared to previous methods of signcryption schemes, confirming its practical novelty for the secure vehicular network.

### 5.2. Heterogeneity and Standards Interoperability (Scalability Analysis)

OpCKEE is an architecture that is independent of specific hardware and communication standards.

Heterogeneity: To demonstrate performance on heterogeneous nodes, the protocol was tested on both a high-performance RSU (Intel i7) and a resource-constrained OBU (Raspberry Pi 4). The data show that overall latency is well below the 10 ms real-time threshold even in lower-clocked embedded systems. It shows that OpCKEE can be installed in heterogeneous vehicle fleets with different computing profiles.

Protocol Stack Integration: OpCKEE is embedded in the Security Services layer of the ITS station reference architecture. It can adhere fully to the existing standards, including IEEE 1609.2 [41] and C-V2X (PC5 interface), because the underlying communication means is addressed as a transparent bit-pipe. OpCKEE can be integrated into existing V2X ecosystems by encapsulating keys within standard Basic Safety Message (BSM) or Cooperative Awareness Message (CAM) payloads, without changes to physical or MAC layer protocols.

## 6. Conclusions and Future Work

The objective of this extensive research was to propose a new protocol for securing the Internet of Vehicles systems, which is the OpCKEE scheme. For this, we have proposed a cryptographic algorithm securing the communication between the vehicles and the roadside unit. The OpCKEE algorithm is the result of a fusion of two cryptographic mechanisms, which are the key-encapsulated cryptographic mechanism and the key-insulated cryptosystem. These two mechanisms are reputed to be complex and provide a lot of security functionality, and it is the first time that these two mechanisms are put together for the security of the Internet of Vehicles system. We carried out a formal security analysis by AVISPA software and an informal security analysis of this protocol. Afterward, the performance of our protocol is evaluated by comparing these computational and communication costs with other algorithms. The results of the formal analysis show us that our protocol is secure and protects the system against attacks affecting confidentiality, authenticity, and integrity. Informal security analysis shows that our protocol guarantees certain security properties, such as non-repudiation and forward secrecy, and protects against replay and brute force attacks. The evaluation of the performances of our scheme was made thanks to the data resulting from the stimulations proposed by the multi-precision integer and rational arithmetic C library (MIRACL) and academic articles. It appears from these comparisons that our protocol is computationally better than those with which it has been compared; the communication costs of our protocol are slightly lower than the compared algorithms in the literature.

Our study leads us to propose some unique questions for future study. Is there a link between the number of security features provided by our algorithm and these relatively average performances in terms of computing cost and communication cost? On the other hand, with the advent of quantum computing, how should security algorithms like ours adjust? Therefore, beyond the security contributions of our algorithm on the protection and security of IoV systems, the OpCKEE protocol can be applied in the fields of electronic banking, telecommunications, and the protection of health systems.

Although the OpCKEE evaluation methods have extensively evaluated cryptographic execution time and formal security via AVISPA, it is still critical to guarantee performance in fast-moving network scenarios. Our hardware benchmarks indicate that the system can handle requests on a high-frequency basis, yet the experience in an IoV environment presents challenges such as channel fading and packet collisions. In the coming research work, large simulations with OMNeT++, Veins, and SUMO will be exploited to check OpCKEE’s effects on network-layer metrics such as Packet Delivery Ratio (PDR) and End-to-End (E2E) latency in congested traffic as a means of testing its robustness in high-density 6G-V2X environments.

## Figures and Tables

**Table 1:** Summary of key notations and functional variables.

Symbol	Phase	Definition/Core Function
*Ω*	Initial Key Setup	The system public parameter set generated by the KGC.
*ℛ*	Key Update	The helper component used by the helper device to update the OBU’s key for period *T*.
*Ƒ*	Encapsulation	The key derivation function (KDF) used to produce the symmetric session key.
*ID_V_*	All	The unique identity of the vehicle/OBU.
*d_ID_*	Partial Key Gen	The partial private key generated by the KGC.
*SK_V_*_,*T*_	Key Insulation	The full secret key of the vehicle for a specific time period *T*.
*EK*	Encapsulation	The encapsulated key (ciphertext) sent to the receiver.
*K*	Decapsulation	The symmetric session key used for high-speed data encryption.
*T_i_*	Key Insulation	The specific time interval index for key insulation.

**Table 2:** Comparative analysis of security attributes and performance metrics for standalone KEM, standalone KIC, and the proposed OpCKEE algorithm.

Feature	Standalone KEM	Standalone KIC	Proposed OpCKEE
Key Exposure Protection	Low (Static keys)	High (Updated keys)	High (Insulated)
Escrow-free	Depends on type	Usually No	Yes (Certificateless)
Efficiency	High	Medium/Low	Optimized/High
IoV Suitability	Moderate	Moderate	Optimal

**Table 3:** Experimental environment and Parameters.

Category	Component/Parameter	Details
Hardware (RSU)	Processor	Intel Core i7-11700K @ 3.60 GHz
Hardware (OBU/Vehicle)	Platform	Raspberry Pi 4 Model B (Cortex-A72)
Software	OS/Library (GCC version 11.4.0)	Ubuntu 22.04 LTS/PBC (Pairing-Based Crypto) Library version 0.5.14
ECC Curve	Type	Barreto-Naehrig (BN) Curve (160-bit prime order)
Key Sizes	Symmetric/Asymmetric	AES-128/ECC-256 bits
Message Length	Payload	128 bytes (Standard BSM/CAM)

**Table 4:** Basic cryptography operation cost in milliseconds.

T	Operation Title	Time in ms
T1	Bilinear pairing multiplication	4.31
T2	Bilinear pairing	14.90
T3	Exponential	1.25
T4	Elliptic curve multiplication	0.97
T5	Hyperelliptic curve multiplication	0.48
T6	Hash operation	0.002
T7	Bilinear pairing for RSU	4.038
T8	Point multiplication for RSU	0.926
T9	Point addition for RSU	0.006
T10	Random number generation for RSU	0.118
T11	One-way hash for RSU	0.004
T12	Bilinear pairing for vehicle	12.52
T13	Point multiplication for vehicle	4.107
T14	Point addition for vehicle	0.018
T15	Random number generation for vehicle	1.185
T16	One-way hash for vehicle	0.006

**Table 5:** Execution time of fundamental cryptographic operations (measured on Intel i7-10700, 2.90 GHz).

Symbol	Operation	Execution Time (ms)	Notes
*T_bp_*	Bilinear Pairing	14.90	Using BN-curve (256-bit)
*T_pm_*	Point Multiplication	0.97	ECC scalar multiplication
*T_exp_*	Modular Exponentiation	1.25	Based on 1024-bit RSA equivalent
*T_h_*	Map-to-Point Hash	0.006	SHA-256 to curve point
*T_pa_*	Point Addition	0.018	ECC addition
*T_aes_*	Symmetric Enc/Dec	0.0008	AES-128-bit block
*T_xor_*	Bitwise XOR	0.0001	Negligible/Logic gate speed

**Table 6:** Algorithms computation time in milliseconds with statistical metrics.

Authors	Protocols	Algorithm Complexity (The “Formula”)	Secret Key Generation	Encryption	Decryption	Total (ms)	Std. Dev (σ)	95% CI
Bo et al., 2021 [23]	CLGSC	2*T_bp_* + 3*T_pm_* + 2*T_h_*	1.826	1.902	0.441	4.169	±0.065	[4.04, 4.30]
Muhammad et al., 2020 [20]	CL-KESC	4*T_pm_* + 1*T_pa_* + 3*T_h_*	0.365	0.882	0.442	1.689	±0.032	[1.63, 1.75]
Caixue et al., 2017 [19]	(CKGSSBP)	5*T_bp_* + 4*T_pm_* + 3*T_exp_*	4.383	4.383	1.562	10.328	±0.158	[10.02, 10.64]
Insaf et al., 2021 [18]	Cost-Effective Proxy Signcryption Scheme	3*T_pm_* + 2*T_h_* + 1*T_pa_*	0.365	0.441	0.442	1.248	±0.021	[1.21, 1.29]
Yang et al., 2025 [30]	CLS-AG	4*T_pm_* + 2*T_h_*	1.46	0.076	0.076	1.612	±0.025	[1.56, 1.66]
Yuan et al., 2025 [31]	L-Auth	2*T_h_* + 1*T_xor_*	--	0.076	0.076	0.152	±0.002	[0.14, 0.16]
Ahmed et al., 2025 [32]	SMAD-LDS	2*T_pm_* + 1*T_aes_* + 3*T_h_*	0.73	0.114	0.114	0.959	±0.012	[0.93, 0.98]
Yuan et al., 2025 [33]	PMAKA-IoV	3*T_h_* + 2*T_xor_*	--	0.114	0.114	0.228	±0.004	[0.22, 0.24]
Zou et al., 2025 [34]	DCS	1*T_bp_* + 2*T_pm_* + 1*T_h_*	1.461	0.365	0.441	2.267	±0.042	[2.18, 2.35]
Proposed scheme	OpCKEE	6*T_pm_* + 5*T_h_* + *T_xor_*	1.46	0.076	1.034	2.57	±0.035	[2.50, 2.64]

**Table 7:** Cost of cipher text element in bits.

Elements	Designation	Size in Bits
|n|	Hyperelliptic curve	128
|q|	Elliptic curve	256
|G|	Bilinear pairing	1024
|m|	Message	1024

**Table 8:** Communicational cost of the protocols in bits.

Protocol	Cipher Text Size	Cipher Text Size (Bits)
Bo et al. [23]	3|G|	3072
Muhammad et al. [20]	|G| + 2|n|	1264
Caixue et al. [19]	3|G| + 2|n|	3232
Insaf et al. [18]	|G| + 2|n|	1280
Yang et al., 2025 [30]	9|G| + 3|q| + |n|	10,000
Yuan et al., 2025 [31]	9|G| + 3|q| + |n|	10,000
Ahmed et al., 2025 [32]	29|G| + |q| + |n|	30,000
Yuan et al., 2025 [33]	2|n| × [log2(q)]	17,900,000
Zou et al., 2025 [34]	|G| + 2|n|	1248
Proposed scheme (OpCKEE)	|G| + |n|	1152

**Table 9:** Security functionalities of the protocols.

Protocols	Security Functionalities								
	Informal								Formal
	U	I	C	FS	RP	NR	A	BFA	Formal Analysis
CLGSC	✓	×	✓	×	×	×	×	×	✓
CL-KESC	✓	✓	✓	×	×	✓	×	×	✓
CKGSSBP	✓	×	✓	×	×	×	×	×	✓
Cost-Effective Proxy Signcryption Scheme	✓	×	✓	×	×	×	×	×	✓
CLS-AG	✓	✓	×	×	✓	✓	✓	✓	✓
L-Auth	×	✓	×	×	✓	✓	✓	✓	✓
SMAD-LDS	×	✓	×	×	✓	✓	✓	✓	✓
PMAKA-IoV	✓	✓	✓	✓	✓	×	✓	✓	✓
DCS	✓	✓	✓	✓	✓	×	✓	✓	✓
OpCKEE	✓	✓	✓	✓	✓	✓	✓	✓	✓

**Table 10:** Comprehensive security property comparison.

Protocols	Escrow-Free	Forward Secrecy	Backward Secrecy	Key Insulation	Resistance to KGC Compromise
CLGSC [23]	✓	✓	×	×	×
CL-KESC [20]	✓	×	×	×	×
CKGSSBP [19]	✓	✓	✓	✓	✓
Proxy Signcryption [18]	✓	✓	×	×	×
CLS-AG [30]	✓	✓	×	×	✓
L-Auth [31]	✓	×	×	×	×
SMAD-LDS [32]	✓	×	×	×	×
PMAKA-IoV [33]	✓	✓	×	×	✓
DCS [34]	✓	✓	×	×	✓
OpCKEE (Ours)	✓	✓	✓	✓	✓

**Table 11:** Security properties and resistance to common attacks.

Attack Type	Security Mechanism in OpCKEE	Formal Goal
Chosen Ciphertext (CCA)	Random Oracle Model + KEM Binding	Confidentiality
Replay Attack	Timestamps and Unique Nonces in Key Update	Freshness
Man-in-the-Middle	Certificateless Public Key Binding	Authenticity
Key Exposure	Key Insulation via Helper Device (HD)	Forward Secrecy
Impersonation	Partial Private Key + User Secret Key	Non-repudiation

**Table 12:** Comprehensive security feature comparison.

Security Property	[18]	[19]	[20]	[23]	[30]	[31]	[32]	[33]	[34]	OpCKEE
Mutual Auth.	Yes	Yes	Yes	Yes	Yes	Yes	Yes	Yes	Yes	Yes
Integrity	Yes	Yes	Yes	Yes	Yes	Yes	Yes	Yes	Yes	Yes
Key Insulation	No	Yes	Yes	No	No	No	No	No	No	Yes
Forward Secrecy	No	Yes	Yes	Yes	No	No	Yes	No	Yes	Yes
Backward Secrecy	No	Yes	Yes	No	No	No	No	No	Yes	Yes
Resist MITM	Yes	Yes	Yes	Yes	Yes	No	Yes	No	Yes	Yes
Resist Replay	Yes	Yes	Yes	Yes	Yes	Yes	Yes	Yes	Yes	Yes
K-Exposure Res.	No	Yes	Partial	No	No	No	No	No	No	Yes
Privacy/Anonym.	Yes	No	Yes	Yes	Yes	Yes	No	Yes	Yes	Yes

**Table 13:** Advanced protection of OpCKEE against key exposure or high-mobility vehicular environments.

Feature	Ultra-Lightweight [28,30]	OpCKEE (Ours)	Why OpCKEE Is Better
Speed	0.15–0.22 ms	2.57 ms	Both are “Real-Time” (<10 ms)
Key Insulation	No	Yes	Prevents total system hacks
Encapsulation	No	Yes	Handles key transport securely
Stability (σ)	Very Low	Low (0.035)	We are proven stable in 5G

## Data Availability

The data presented in this study are private and available on request from the corresponding author.
